# Circulating Tumor Cell Detection and Polyomavirus Status in Merkel Cell Carcinoma

**DOI:** 10.1038/s41598-020-58572-9

**Published:** 2020-01-31

**Authors:** Magali Boyer, Laure Cayrefourcq, Françoise Garima, Vincent Foulongne, Olivier Dereure, Catherine Alix-Panabières

**Affiliations:** 1 Laboratory of Rare Human Circulating Cells, University Medical Centre of Montpellier, 34093 Montpellier, France; 2 Pathogenesis and Control of Chronic Infections, University of Montpellier, INSERM, EFS, University Medical Centre, 34090 Montpellier, France; 3 Department of Dermatology, University Medical Centre of Montpellier and INSERM 1058 Pathogenesis and Control of Chronic Infections University of Montpellier, 34090 Montpellier, France

**Keywords:** Skin cancer, Skin cancer

## Abstract

The incidence of Merkel cell carcinoma (MCC), a rare and highly metastatic skin malignancy, has sharply increased in the last decade. Clinical biomarkers are urgently needed for MCC prognosis, treatment response monitoring, and early diagnosis of relapse. The clinical interest of circulating tumors cells (CTCs) has been validated in many solid cancers. The aim of this study was to compare CTC detection and characterization in blood samples of patients with MCC using the CellSearch System and the RosetteSep -DEPArray workflow, an innovative procedure to enrich, detect and isolate single CTCs. In preliminary experiments (using spiked MCC cell lines) both methods allowed detecting very few MCC cells. In blood samples from 19 patients with MCC at different stages, CellSearch detected MCC CTCs in 26% of patients, and the R-D workflow in 42% of patients. The detection of CTC-positive patients increased to 52% by the cumulative positivity rate of both methodologies. Moreover, Merkel cell polyomavirus DNA, involved in MCC oncogenesis, was detected in tumor biopsies, but not in all single CTCs from the same patient, reflecting the tumor heterogeneity. Our data demonstrate the possibility to detect, isolate and characterize CTCs in patients with MCC using two complementary approaches.

## Introduction

Merkel Cell Carcinoma (MCC) is a rare and aggressive neuroendocrine skin cancer, the incidence of which has been steadily increasing over the last decades^1,2^. It is one of the most lethal skin malignancies after melanoma^3^, and more frequently affects fair-skinned men with a median age of 70 years at diagnosis^4^. MCC usually appears as a rapidly growing red or purple nodule mostly located on UV-exposed areas (head, neck or upper limbs). Several risk factors have been identified, such as UV exposure, disease- or treatment-related immunosuppression, notably transplanted and HIV-infected patients^5^. A new virus belonging to the *Polyomaviridae* family and named Merkel cell polyomavirus (MCPyV) has been identified in some MCC tissue specimens^6^. The clonal integration of the viral DNA in the genome of MCC cells^7^ suggests that this phenomenon is an early event occurring before malignant transformation^8^. This virus is present in most MCC (about 80% of patients) and seems to play a direct role in malignant transformation, most notably through the intervention of oncogenic proteins^6^. Indeed, MCPyV expresses the large T antigen and the small T antigen that display a strong oncogenic activity^9,10^. These oncogenic viral proteins are both expressed in MCPyV^+^ MCC and seem to be necessary for the maintenance of MCPyV^+^ MCC cell lines^11^. Conversely, MCPyV^−^ MCC are characterized by higher number of mutations in key genes, a UV-mutational signature, and more chromosomal aberrations compared with MCPyV^+^ tumors^8,12^, suggesting two distinct oncogenic pathways.

Circulating tumor cells (CTCs) are considered as the real-time *liquid biopsy* for patients with cancer, described for the first time in 2010^13,14^. The stem-cell properties and sometimes the clustering capacities of the most aggressive CTCs are related to metastasis progression^15,16^. CTC detection and characterization may provide information on the cancer progression, prognosis, and therapy response. Indeed, numerous clinical studies and meta-analyses, including in large cohorts of patients, have shown that CTC number is an important indicator of the risk of progression or death in patients with metastatic solid cancer (e.g., breast, prostate, colon cancer)^17–19^. Other studies demonstrated that CTC number decreases in patients who respond to cancer therapy^20–22^, whereas it increases in poor responders. In MCC, liquid biopsy and CTCs could be used to obtain information about the oncogenic pathway in this poorly understood malignancy. For example, we can follow the viral status, and the evolution of mutational burden in serial liquid biopsies compared to the initial tissue biopsy. Up to now, few studies have investigated the clinical relevance of biomarkers in MCC. One study correlated the presence of miR-375 in serum of patients with MCC^23^, some others determined T antigen antibodies as a prognostic marker in MCC^24,25^ and only three studies have investigated CTC detection in MCC: two based on EpCAM-positive selection of CTCs using the CellSearch system and one using the Maintrac system^26–28^. These three studies found that CTC detection in MCC is feasible, and one also reported that the presence of CTCs is a prognostic factor of worse clinical outcome^28^. As the biology of MCC CTCs is not yet fully understood, we decided to detect CTCs without any bias of selection for the enrichment step.

Thus, we describe in this study a new workflow based on negative enrichment of MCC CTCs using the RosetteSep technology combined with CTC detection and sorting with the DEPArray technology. We subsequently tested blood samples from 19 patients with MCC using this new workflow and the CellSearch system, and correlated the CTC detection with biological, pathological and clinical data. In addition, we investigated the MCPyV status in single CTCs, and compared the results with the viral status of the corresponding primary or metastatic tumor biopsies. We describe in this study two technologies for CTC detection in MCC and MCPyV detection at single cell level in order to develop tools to better understand the biology of this cancer.

## Results

### Phenotypic characterization of Merkel cell carcinoma (MCC) cell lines

**Figure 1:**
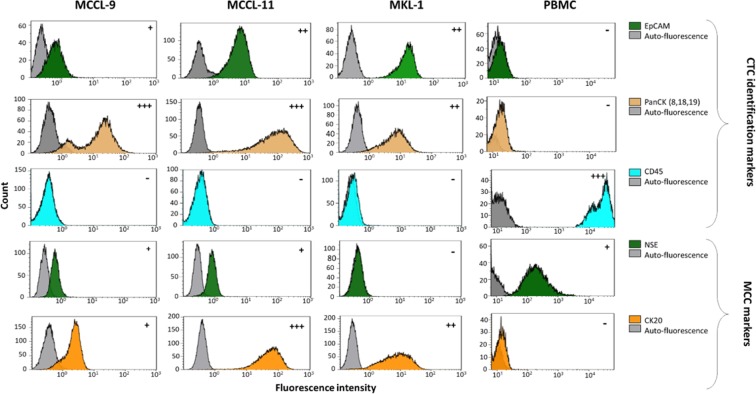
Phenotypic characterization of MCC cell lines. Specific CTC, leukocyte and MCC markers were used to characterize the MCCL-9, MCCL-11 and MKL-1 Merkel cancer cell lines and PBMCs as controls. The markers presented in this Fig. are those that have been selected to detect CTCs in MCC. (+) represents a positive marker, and the number of (+) indicates the signal intensity. (−) represents a negative marker. Grey represent the auto-fluorescence of the MCC cell lines (negative control). All these markers were also tested on PBMCs to determine which markers can be used to discriminate CTCs from normal blood cells. *Abbreviations:* EpCAM: Epithelial Cell Adhesion Molecule, CK: Cytokeratin. Pan: panel. NSE: Neuron-Specific Enolase, CD: Cluster Differentiation.

To select markers that could be used to identify circulating MCC cells in blood samples, first we determined the phenotype of three MCC cell lines (MCCL-9, MCCL-11 and MKL-1) using markers that are commonly employed for the histopathological diagnosis of this cancer (Neuron-Specific Enolase (NSE); Synaptophysin, Chromogranin A; Cytokeratin 20 (CK20); and CD56) and markers usually used for CTCs detection (Epithelial Cell Adhesion Molecule (EpCAM); PanCK (8, 18, 19); Vimentin; CD24, CD44, CD45) (Fig. 1 and Supplementary Fig. S1). All markers were first tested independently.

Concerning the MCC-specific markers, all three cell lines expressed CD56, chromogranin A and CK20, but not synaptophysin. The MCCL-9 (MCPyV^−^ cell line) and MCCL-11 (MCPyV^+^) expressed NSE. However, the antibodies against chromogranin A and CD56 labeled also blood cells (Supplementary Fig. S1), precluding their use to detect circulating MCC cells in blood samples. The three MCC cell lines also expressed some of the markers used for CTC detection, particularly the cytokeratin panel (8, 18, 19) and the Epithelial Cell Adhesion Molecule (EpCAM), that is not expressed by the PBMCs **(**Supplementary Fig. S2**)**. As expected, the three MCC cell lines did not express the hematopoietic marker CD45 and vimentin, a marker commonly used for mesenchymal CTC detection but also expressed in all leukocytes. Concerning CD24 and CD44, used to identify the cancer stem cells^29,30^, MCCL-9 and MCCL-11 cells were CD24^−^/CD44^+^, whereas the MKL-1 cell line displayed a CD24^+^/CD44^−^ profile.

On the basis of these results, we selected EpCAM, CK20 and NSE to differentiate circulating MCC cells from normal blood cells, and CD45 as exclusion marker. We tested all the antibodies against the selected markers together to ensure that binding/detection was not hampered by any steric obstruction (Supplementary Fig. S3).

### Detection of MCC cells in spiking experiments

To test whether these markers could identify MCC cells in blood, we spiked a known number of MCCL-9, MCCL-11, or of MKL-1 cells into blood of different healthy donors to mimic blood samples from patients with MCC. We detected MCC cells in these blood samples using the CellSearch system (an EpCAM-positive enrichment method based on magnetic beads selection combined with an immunocytochemical staining to identify CTCs), and the new workflow that combines RosetteSep for negative enrichment, by forming a network of unwanted blood cells discarded after blood centrifugation on a density medium, combine with the DEPArray, a dielectrophoresis technology allowing visual selection and single cell sorting, for cell identification. At least three experiments were done for each cell lines with each detection methods.

#### MCC CTC detection using the CellSearch system

**Figure 2:**
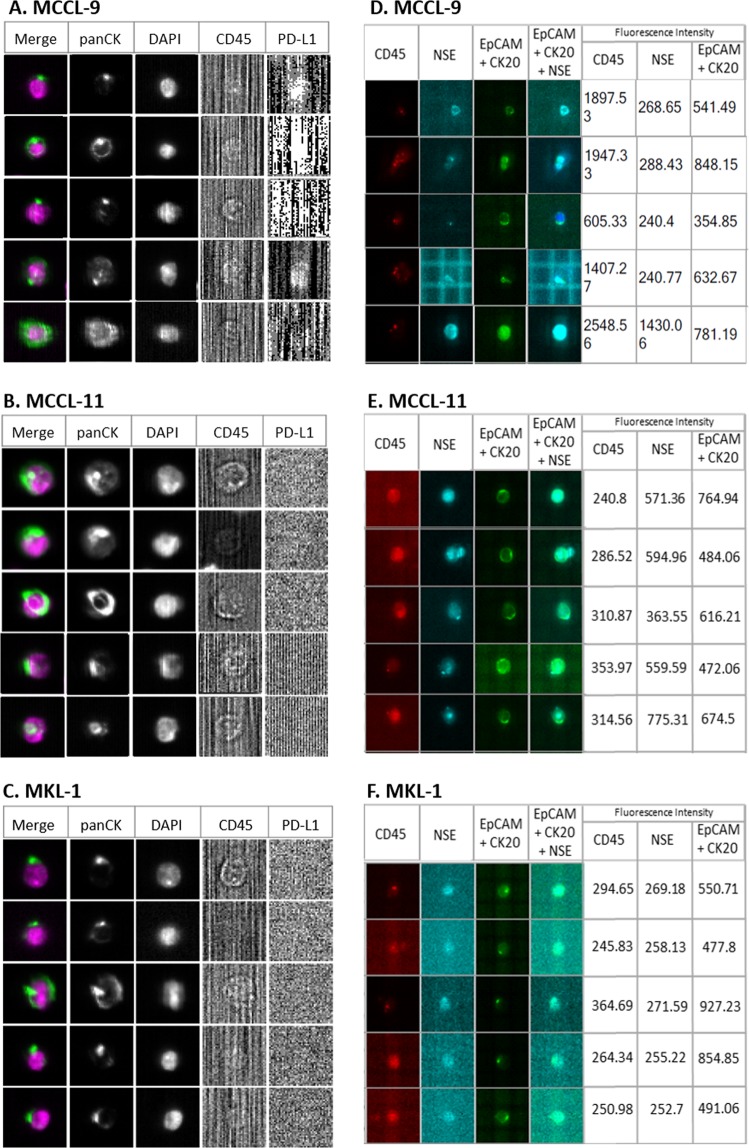
Representative photos of MCC cells detected in spiking experiments. Detection of **(A)** MCCL-9, **(B)** MCCL-11 and **(C)** MKL-1 cells added to blood from healthy donor using the CellSearch system; an anti-PD-L1 antibody was added in the fourth channel for CTC characterization in the CellSearch system. Detection of **(D)** MCCL-9, (**E**) MCCL-11 and **(F)** MKL-1 cells added to blood sample from healthy donors using the “RosetteSep–DEPArray” workflow; anti-CD45 conjugated to PE, anti-NSE conjugated to APC, anti-EpCAM conjugated to FITC and anti-CK20 conjugated to FITC antibodies were used for CTC detection. In DEPArray, fluorescence intensities below 400 were considered negative, according to manufacturer’s instructions. *Abbreviations:* EpCAM: Epithelial Cell Adhesion Molecule, CK: Cytokeratin. Pan: panel. NSE: Neuron-Specific Enolase.

**Figure 3:**
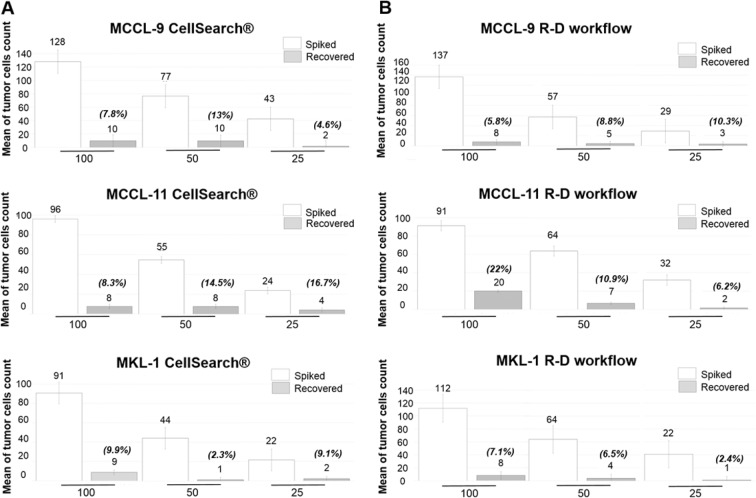
Number and percentages of tumor cells recovered using the CellSearch system (**A**) and the ‘RosetteSep-DEPArray’ workflow (**B**). Different numbers of MCCL-9, MCCL-11 and MKL-1 cells (100, 50 and 25) were spiked in blood of healthy donors (mean of 3 different experiments). The exact number of spiked tumor cells has been verified and used for the calculation of the recovery rate.

We determined the CellSearch system recovery rate by spiking 100, 50 and 25 MCCL-9, MCCL-11 or MKL-1 cells in 7.5 mL of blood (Fig. 2A–C). Although, 50 to 70% of spiked cells were selected as potential events to review by the CellSearch software, we could identify as tumor cells only 8.5% of MCCL-9, 13.1% of MCCL-11 and 8.8% of MKL-1 cells by strictly following the CellSearch instructions according to which a CTC is a cell with cytokeratin staining larger than the nucleus (DAPI) (Fig. 3A). Specifically, we detected 7.8%, 13% and 4.6% of MCCL-9 cells (n = 3), 7.7%, 13.7% and 18% of MCCL-11 cells (n = 3) and 10.3%, 4.5% and 11.5% of MKL-1 cells (n = 3) in the blood samples when 100, 50 or 25 MCC cells were spiked in, respectively. Indeed, as already described in the literature, the cytokeratin network in MCC cells can be observed as a dot^27^. When following carefully the CellSearch’ instructions, these events cannot be identified and validated as CTCs, which could explain the few numbers of CTCs detected in spiking experiments. An adaptation of CellSearch cassette reading should be necessary to obtained better recovery rates. Moreover, as anti-PD-L1 immunotherapy is the newly FDA-approved treatment for metastatic MCC^31^, we defined the status for PD-L1 expression of the three MCC cell lines based on our optimized protocol already described in Mazel *et al. Mol Oncol* 2015^32^. The addition of an anti-PD-L1 antibody in the 4^th^ channel of the CellSearch system indicated that the three MCC cell lines did not express PD-L1.

#### CTC detection using the RosetteSep–DEPArray (R-D) workflow

**Figure 4:**
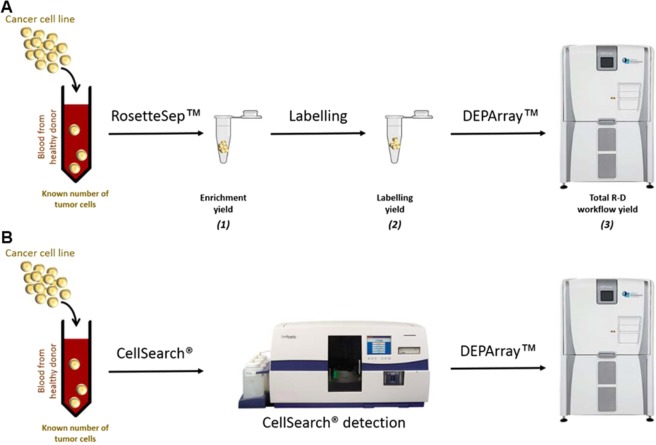
Illustration of RosetteSep-DEPArray workflow steps and CellSearch-DEPArray combination. (**A**) R-D workflow. A known number of tumor cells (Merkel cancer cell line) have been spiked in the blood of healthy donors. The recovery rate of tumor cells (yield) were calculated at each step of this R-D workflow: we counted the remaining tumor cells in the sample (*1*) after the enrichment step (RosetteSep), (2) after the labelling step and (3) after the detection step (DEPArray) and compared this counting with the initial number of spiked tumor cells. (**B**) CellSearch-DEPArray. CTCs are detected using the CellSearch system and the sample is taken from the cassette of the CellSearch to subsequently sort single CTCs with the DEPArray.

For the R-D workflow, we added the same numbers of MCCL-9, MCCL-11 and MKL-1 cell lines to 10 mL healthy donor blood samples. As this procedure has not been validated yet (differently from the CellSearch system), we evaluated each step of the workflow. (Fig. 4) First, we determined the enrichment yield as the number of calcein-labeled cells (63% for MCCL-9, 74% for the MCCL-11- and 53% for the MKL-1-spiked samples; n = 3). Then, we incubated the enriched cells with the antibody cocktail described in Material and Methods to determine the labeling yield (43.6% for MCCL-9, 58% for MCCL-11 and 60% for MKL-1 cells).

After the RosetteSep enrichment step, we loaded labeled cells in DEPArray cartridge. The three MCC cell lines were detected by DEPArray (Fig. 2D–F**)**. Cells identified as tumor cells were EpCAM and/or CK20 (+) (FITC) (fluorescence intensity > 400), NSE (+/−) (APC), DAPI (+) and CD45 (−) (PE). The three cell lines were positive for CK20/EpCAM and negative for CD45 (exclusion marker, specific of leukocytes) and only MCCL-9 and MCCL-11 were positive for NSE. As observed with CellSearch, CK staining was displayed as a dot in MCC cell lines. With the R-D workflow, we recovered 8.3% of MCCL-9, 13.3% of MCCL-11 and 5.3% of MKL-1 cells (Fig. 3B). Specifically, we detected 5.8%, 8.8%, 10.3% of MCCL-9 (n = 3), 22%, 10.9% and 6.2% of MCCL-11 cells (n = 3), and 7.1%, 6.5% and 2.4% of MKL-1 cells (n = 3) in the samples with 100, 50 and 25 MCC cells, respectively. The estimated detection threshold of the R-D workflow was about 25 cells per 10 mL of blood (Fig. 3B). Moreover, blood samples from healthy donors (n = 3) were tested with this new workflow and no CTCs were detected.

In conclusion, both methods (CellSearch and R-D workflow) showed comparable recovery rates for tumor cell detection. However, only the R-D workflow allowed the direct cell sorting after the detection step.

### MCPyV status of single tumor cells – Proof of concept using MCC cell lines

After the CellSearch analysis or the negative enrichment with RosetteSep, MCC cells were sorted with the DEPArray as single cells or as a pool. Then, we determined the presence of MCPyV DNA using genomic DNA obtained by Whole Genome Amplification (WGA) of single MCC cells with the *Ampli*1 WGA kit (MENARINI SILICON BIOSYSTEMS). Quantitative PCR (qPCR) analysis with primers that target the VP2/3 region of MCPyV confirmed that MCPyV DNA was present in MKL-1 and MCCL-11 but not in the MCCL-9 cells. The qPCR targeting VP2/3 of MCPyV was compared with a qPCR targeting the C-terminal part of the large T antigen of MCPyV. We could observe that both methods detect MCPyV in DNA of MCCL-11 and MKL-1 cell line from DNA extraction, and not in MCCL-9 cell line. However, the MCPyV was detected in DNA of single cell of MCCL-11 and MKL-1 only with the qPCR targeting the VP2/3 region of MCPyV. This data demonstrates that single MCC cells can be used to assess the MCPyV status with the qPCR targeting VP2/3.

### Patients’ characteristics

To test whether these two methods could detect MCC CTCs, we used 28 blood samples from 19 patients with stage I/II (n = 8; 42%), stage III (n = 7; 37%), and stage IV MCC (n = 4; 21%). The primary tumor site was on head and neck (n = 7), inferior limb (n = 5), upper limb (n = 2) and trunk (n = 2), and was unknown in 2 patients. All patients underwent wide surgical excision of the primary tumors followed by radiotherapy. Five patients (26%) had a history of other diseases (basal-cell carcinoma, papillary thyroid carcinoma, breast carcinoma, glaucoma, adenoma). Three of the four patients with stage IV MCC were treated with systemic chemotherapy and one with immunotherapy against PD-1 at the time of blood collection. One patient with stage I MCC was on long-term immunosuppressive treatment after kidney transplant (patient 2). Sequential blood samples were available for four patients (n = 2 to 6 blood samples at different times).

### CTC detection in blood samples from patients with MCC

**Figure 5:**
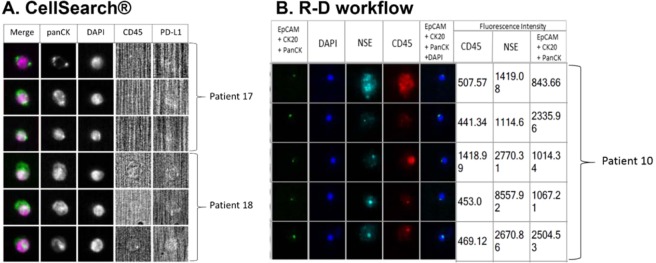
Representative images of CTCs from three patients with MCC. CTCs were detected using (**A**) the CellSearch system in blood samples of patients 17 and 18; CTCs are clearly PD-L1 negative (B7H1 antibody: 3 µg); (**B**) Analysis of a blood sample from patient 10 with the ‘RosetteSep-DEPArray’ workflow using the anti-CD45 conjugated to PE, anti-NSE conjugated to APC, anti-EpCAM conjugated to FITC, anti-Pan CK conjugated to FITC, and anti-CK20 conjugated to FITC antibodies. Fluorescence intensities below 400 were considered negative, according to the manufacturer’s instructions. Cytokeratin expression is detected as a dot in CTCs of patients with MCC, as described by Blom *et al*.^27^. *Abbreviations:* EpCAM: Epithelial Cell Adhesion Molecule, CK: Cytokeratin. Pan: panel. NSE: Neuron-Specific Enolase.

We could detect CTCs in blood samples from five patients with MCC (26% of 19) using the CellSearch system and from eight patients (42% of 19) with the R-D workflow (Table 1) and (Fig. 5). Only three patients (16%) were positive with both methods. Analysis of the correlation between clinical data and CTC presence (Table 1) showed that CTC detection with the CellSearch system correlated significantly (p < 0.05) with MCC stage (M_0_ *vs* M_1_). CTC detection with both methods (CellSearch and R-D workflow) was significantly associated with the number of metastases (≤3 *vs* >3) in patients with stage IV MCC, indeed CTCs were detected in 75% of stage IV MCC patients. Comparison of the individual results suggested that the two detection methods identified different CTC sub-populations. For example, in the six blood samples of patient 17 (during chemotherapy), the CellSearch system identified more than 1000 CTCs, while the R-D workflow detected at most 18 CTCs (Table 2). Moreover, the number of CTCs detected in the subsequent samples increased with the R-D workflow (from 1 to 18), but not with the CellSearch system. For patient 10, three blood samples were collected at different time points (two before chemotherapy initiation, and one after treatment and before primary tumor surgery). The R-D workflow detected CTCs only in the first two samples (17 and 23 CTCs per blood sample), but not in the third sample after treatment. Moreover, in five patients (Patients 2, 3, 10, 12 and 13), the R-D workflow could detect up to 23 cells, whereas the CellSearch system did not detect any (Table 2). By cumulating the positivity rate of both technologies, CTC detection rate increased to 52% (10/19). Finally, all CTCs detected with the CellSearch system were PD-L1 negative.

**Table 1:** Clinico-pathological characteristics and CTC detection in patients with MCC.

	Combined					R-D Workflow					CellSearch system				
Clinicopathological parameter	CTC positive ≥ 1		CTC negative		*p* value	R-D-CTC positive ≥ 1		R-D-CTC negative		*p* value	CS CTC positive ≥ 1		CS CTC negative		*p* value
Total patients	*n*	%	*n*	%		*n*	%	*n*	%		*n*	%	*n*	%	
19	10	53	9	47		8	42	11	58		5	26	14	74	
**Age, years**					1					0.369					1
≤75	5	26	4	21		5	26	4	21		2	10	7	37	
>75	5	26	5	26		3	16	7	37		3	16	7	37	
Mean	75.1														
Median	76														
**Sex**					0.369					0.352					0.602
Men	7	37	4	21		6	32	5	26		2	10	9	47	
Women	3	16	5	26		2	10	6	32		3	16	5	26	
**AJCC stage**					0.607					0.292					0.07
I/II	3	16	5	26		2	10	6	32		1	5	7	37	
III	4	21	3	16		3	16	4	21		1	5	6	32	
IV	3	16	1	5		3	16	1	5		3	16	1	5	
**M0/M1**					0.582					0.262					**0.03**
M0	7	37	8	42		5	26	10	53		2	10	13	68	
M1	3	16	1	5		3	16	1	5		3	16	1	5	
**Nbr organs with metastasis**					0.210					**0.05**					**0.01**
≤3	7	37	9	47		5	26	11	58		2	10	14	74	
>3	3	16	0	0		3	16	0	0		3	16	0	0	
**Cancers antecedents**					1					0.894					1
Yes	4	21	1	5		2	10	3	16		1	5	4	21	
No	7	37	7	37		6	32	8	42		4	21	10	53	

n: number of patients; %: percentage of patients; *p* values calculated with the Fisher test. R-D: RosetteSep – DEPArray workflow; CS: CellSearch; p ≤ 0.05.

**Table 2:** CTC detection in patients with MCC.

Patients	Cancer stage	Blood collection	Time interval	CellSearch	R-D workflow
1	I	After diagnosis	D_0_	0	0
2	I	After diagnosis	D_0_	0	4
		After diagnosis	D_120_	0	0
3	I	After diagnosis	D_0_	0	1
4	II	After diagnosis	D_0_	0	0
5	II	After diagnosis	D_0_	0	0
6	II	After diagnosis	D_0_	2	0
7	II	After diagnosis	D_0_	0	0
8	II	After diagnosis	D_0_	0	0
9	III	After diagnosis	D_0_	2	0
10	III	After diagnosis	D_0_	NA	17
		Before chemotherapy	D_14_	0	23
		Under treatment	D_141_	0	0
11	III	After diagnosis	D_0_	0	0
		After diagnosis	D_152_	0	0
12	III	After diagnosis	D_0_	0	1
13	III	After diagnosis	D_0_	0	1
14	III	After diagnosis	D_0_	0	0
15	III	After diagnosis	D_0_	0	0
16	IV	Before chemotherapy	D_0_	0	0
17	IV	Before chemotherapy	D_0_	NA	0
		Under treatment	D_78_	NA	1
		Under treatment	D_105_	NA	9
		Under treatment	D_142_	1022	18
		Under treatment	D_147_	881	NA
		After treatment	D_185_	915	NA
18	IV	Under treatment	D_0_	10	6
19	IV	Under treatment	D_0_	32	6

CTC detection was performed using the CellSearch system and the RosetteSep-DEPArray (R-D) workflow in 19 patients. We could get a follow-up for four patients; 28 blood samples in total. D_0_: blood sample at Day 0. The number of CTCs was normalized between the two detection methods.

### Single CTC sequencing

**Figure 6:**
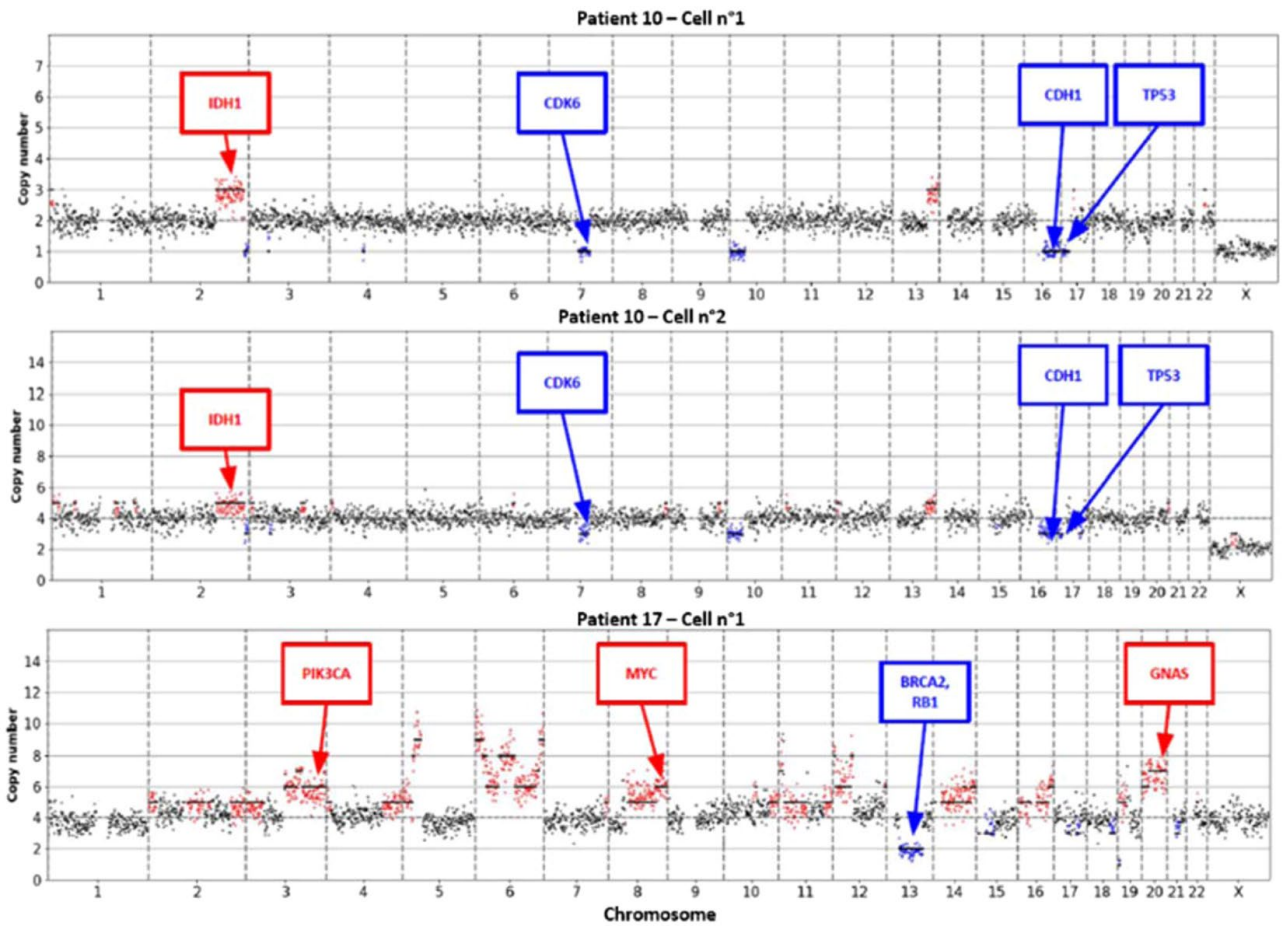
Copy number Aberration (CNA) in four single CTCs. The CNA profile was investigated in four single CTCs (sorted with DEPArray) from three different patients. Three of the four single cells show aberrant chromosome profiles and can be considerate as CTCs.

After the CellSearch and RosetteSep enrichment step, we analyzed cells with DEPArray for single cell sorting; however, around 75% of the CTCs cells identified with the CellSearch system were lost in this process. We then investigated copy number variations in five single CTCs sorted with the R-D workflow to confirm their tumor origin **(**Fig. 6**)**. As in one case DNA quality after WGA was too low to allow proper analysis, we could test only four single CTCs from three different patients with MCC. The two single CTCs from patient 10 displayed the same mutation profile with *TP53*, *CDH1* and *CDK6* deletions and *IDH1* gain. Two of the four samples have a ploidy of 4 (Cell n°2 from patient 10, and Cell n°1 from patient 17) may be explained by a profile identical to that at ploidy 2 recovered along with a normal diploid cell. The single cell from patient 13 had a flat profile with no significative chromosomal aberration, this sorted cell may be a leukocyte. Cell from patient 17 showed clear aberrations, involving several chromosomal regions that encompass cancer-related genes, such as *BRCA2* (deletion) and *PI3KCA* (amplification). These results confirmed the R-D workflow feasibility for detecting and sorting MCC CTCs from blood samples and the possibility of downstream single-cell genomic analyses after genomic DNA amplification.

### Comparison of the MCPyV status in tissue and liquid biopsies

To compare MCPyV status in tumor tissue biopsies from patients positive for CTCs, we collected nine tumor biopsies from eight patients (two tissue biopsies were available for patient 17 of two different metastases). We could detect MCPyV only in seven tumor biopsies because of the poor genomic DNA quality of two virus-negative biopsies. In parallel, we investigated the viral status of single CTCs (Table 3). MCPyV status seems to be heterogeneous in CTCs from the same patient (intra-tumor heterogeneity). For example, in patient 17, among the 52 single CTCs we tested, only 5 (10%) were MCPyV^+^; in patient 18, only one CTC (25%) was MCPyV^+^; and in patient 10, among the 24 tested CTCs, 75% were MCPyV^+^. Overall, up to 75% of the tested CTCs appear as MCPyV^+^. These data suggest that despite the positivity of the tumor tissue biopsies, some single CTCs from the same patient can be MCPyV^−^, highlighting a possible tumor cell heterogeneity (Table 3).

**Table 3:** MCPyV status of single CTCs and the corresponding tissue biopsy (primary tumor or metastases) in eight patients with MCC.

Patients	Tumor Status	Number of CTCs analyzed for the MCPyV status	Number of MCPyV^+^ CTCs	Percentage of MCPyV^+^ CTCs
6	−	0		
9	+	1	0	0%
10	+	24	18	75%
12	+	1	0	0%
13	+	2	0	0%
17 *Metastasis 1*	+	52	5	10%
17 *Metastasis 2*	−			
18	+	4	1	25%
19	+	4	1	25%

## Discussion

*Liquid biopsy* is a potential tool to closely monitor MCC, to identify patients at high risk of recurrence, to detect early clues of relapse, and to predict treatment efficacy. A few studies have addressed this issue in MCC with the quantification of (*i*) antibodies against large T antigen, and (ii) miR-365 in the serum. In addition, CTCs have been included as prognostic markers in MCC. Three recent studies addressed CTC detection in MCC^26–28^, two using positive selection strategy for CTC enrichment before their detection and one using erythrocytes lysis. Conversely, our study compared two different complementary methods for CTC detection in MCC: (*i*) the CellSearch system, the only FDA-cleared technology for metastatic breast, prostate and colon cancer^17–19^, and (*ii*) the RosetteSep and DEPArray (R-D) workflow, where CTC detection is based on negative enrichment (leukocyte depletion) followed by CTC detection by DEPArray that also allows the direct sorting of single CTCs. Our preliminary experiments performed with MCC lines indicate that both methods display similar performances. We have clearly shown that tumor cells in MCC do not express the Cytokeratin (CK) like in other cancer types. Indeed, as shown in our study, CK is frequently detected as a dot and not as a diffuse staining in the whole cytoplasm. We identified and counted CTCs only based on the strict criteria defined by the manufacturer and have missed, in a stochastic way, many of the spiked tumor cells. In our study, we included patients with an advanced disease (locally advanced, stage III, and metastatic, stage IV) as well as local cancer patients (I, II). As this cancer type is rare, the aim was to include first metastatic patients to increase the chance to detect CTCs but also localized cancer patients to assess the performance of our new technology where the cancer should be less disseminated. In experiments performed using blood samples from patients with MCC, the CellSearch system detected CTCs in 26% of patients, and the R-D workflow in 42% of patients. The detection of CTC-positive patients increased to 52% by cumulating the positivity rate of both methodologies. We detected CTCs mostly in patients with stage III/IV MCC, but also in some patients with early-stage MCC (patient 6 with the CellSearch system, and patients 2 and 3 with the R-D workflow). CTC detection was correlated with tumor stage, presence of metastases, and number of organs with metastases.

CTC analysis at different times in four patients suggests that CTC dynamics could be correlated with the clinical outcome. Indeed, in patient 17 (non-responding stage IV MCC), CTC number (R-D workflow) slightly increased over time. In patient 10 (stage III MCC), CTC disappeared after surgery and chemotherapy that led to a reduction of lymph node metastases. However, the very short follow-up (mean: 6 months) did not allow establishing any correlation between CTC detection and clinical outcome (overall survival and progression-free survival).

Moreover, CTC evaluation by the two different methods used in this study provides complementary information. For instance, for patient 17 (stage IV MCC), the CTC yield was much higher with the CellSearch system compared with the R-D workflow. However, overall, the R-D workflow detected CTCs in more samples, probably because it identifies EpCAM^−^ CTCs, a profile that may be overlooked by the CellSearch system. Moreover, sequencing of individually sorted CTCs after the R-D workflow allowed showing specific gene gains and losses that are usually associated with malignancies, thus confirming the malignant nature of circulating cells. Therefore, the two methods are complementary and the use of both technologies in parallel may increase CTC detection rate in patients with MCC. Of note, all CTCs detected with the CellSearch system were PD-L1 negative, although metastatic MCC can be efficiently treated with PD-L1-based immunotherapy. These data suggest a heterogeneity in the primary tumor, metastases and CTCs, as previously described for other solid tumors^33,34^.

Two previous studies on CTC detection in MCC with the CellSearch system identified CTCs in 41% of the tested patients^27,28^. On the other hand, the Maintrac technology, based on the EpCAM/CD56 or CK20 markers for CTC detection, identified CTCs in up to 90% of patients. This very high detection rate was obtained at the cost of specificity because 30% of healthy patients also were considered as CTC-positive with the EpCAM/CK20 co-labelling^26^.

Concerning CTC molecular characterization, DEPArray allowed the isolation of single cells after the RosetteSep- or CellSearch-based enrichment, but with considerable loss of CTCs. After WGA of single CTCs, analysis of copy number aberrations and MCPyV status in such cells showed that tumor tissue biopsies were mainly MCPyV^+^, whereas single CTCs from the same patient showed a more heterogeneous profile for detection of the polyomavirus. Heterogeneity of MCPyV status was already described, notably between primary and metastatic tumors^35^. These discrepancies might be explained by (i) a bias of amplification of the CTC genome, (ii) a loss of viral DNA upon cancer cell detachment and dissemination, or (iii) the presence of MCPyV^−^ clones in metastases, suggested by the hit-and-run theory, as discussed previously^36^. A better understanding of the metastatic cascade biology in MCC is crucial and further insights may be more easily obtained by CTC analysis.

In conclusion, this study confirms the feasibility of CTC-based liquid biopsy in MCC and emphasizes the interest of using two different complementary approaches that may identify different CTC subsets. Although follow-up evaluations are now required to assess the clinical outcome (Progression-Free Survival and Overall Survival), the present study allows investigations on minimal residual disease in MCC patients due to the sufficient capturing of CTCs for enumeration and subsequent characterization. Indeed, gene alterations and MCPyV status can be analyzed in single CTCs, opening new avenues for the molecular characterization of CTCs, of oncogenic events according to the tumor stage, and of tumor heterogeneity as well as for personalized patient monitoring. However, these data must be validated in larger cohorts of patients with MCC, and efforts should focus on developing a reliable, standardized and robust method to detect and enumerate CTCs in MCC. To meet this challenge, a European consortium involving scientists, dermatologists and virologists will help to achieve this goal (e.g., European Liquid Biopsy Society – ELBS).

## Material and Methods

### Cancer cell lines

The MCCL-9 (MCPyV-), and MCCL-11 cell lines (MCPyV+), were derived from metastatic sites^37^ and kindly provided by Prof. Brandner (University Medical Center, Hamburg, Germany). These cancer cell lines grow on a layer of inactive F255 fibroblasts, in RPMI 1640 medium, supplied with 5 mM L-glutamine, 5 mM insulin-transferrin-selenium and 10% fetal calf serum (FCS). The MKL-1 cell line (09111801, Public Health England), derived from a lymph node MCC metastasis, is MCPyV^+^ and was cultured in non-adherent conditions in RPMI 1640 medium, supplemented with 5 mM L-glutamine and 10% FCS.

### Flow cytometry and immunofluorescence experiment

To specifically identify circulating MCC cells, their phenotype was defined by independently testing a large panel of antibodies (Supplementary Table S1) in the three MCC cell lines. Antibodies against surface membrane markers (EpCAM, synaptophysin, CD24, CD44, CD56 and CD45) were directly added to cell samples at room temperature in the dark for 30 minutes. To evaluate the expression of different intracellular biomarkers, cells were fixed and permeabilized with CellSearch kit reagents (ref. 7900001– MENARINI SILICON BIOSYSTEMS) at the same time of antibodies incubation (NSE, vimentin, chromogranin-A, PanCK (8, 18, 19) and CK20), for 30 minutes at room temperature, in the dark. Peripheral blood mononuclear cells were used as negative control. Cell markers were detected with a CyAn ADP Analyzer (BECKMAN COULTER) and data were analyzed with the Kaluza software (BECKMAN COULTER).

The CellSearch system is the only FDA-cleared system for CTC detection in metastatic breast, colon and prostate cancer.

### CTC detection using the CellSearch system

The CellSearch system is the only FDA-cleared system for CTC detection in metastatic breast, colon and prostate cancer^38–40^. This technology is based on the selection and detection of epithelial cells from blood samples by immunomagnetic enrichment and immunofluorescence detection. For this study, the IVD CTC kit (7900001 – MENARINI SILICON BIOSYSTEMS) was used. Briefly, blood samples collected in special CellSave tubes (MENARINI SILICON BIOSYSTEMS) were diluted and centrifuged to be loaded in the CellSearch Autoprep system. This automate performs a magnetic enrichment with an anti-EpCAM antibody coated with magnetic beads and labels the enriched cells with a cocktail of antibodies (anti-Pan CK conjugated to PE, -CD45 conjugated to APC, and DAPI dye) for immunofluorescence detection. Even CTCs with a low expression of EpCAM can be isolated using the CellSearch system^41^. In this study, an anti-human B7H1/PD-L1 monoclonal antibody (Cat N° FAB1561P, R&D SYSTEM) was added to the CellSearch kit to characterize PD-L1 expression in CTCs. The CellSearch cassettes positive for CTC detection were selected and cells were subsequently sorted one by one using the DEPArray System (MENARINI SILICON BIOSYSTEMS).

### RosetteSep-DEPArray workflow for CTC detection

In this study, the RosetteSep-DEPArray workflow, an alternative way to enrich, detect and sort MCC CTCs, was improved. First, a negative selection with the RosetteSep technology (STEMCELL TECHNOLOGIES) was used to enrich CTCs, according to the manufacturer’s instructions. Briefly, RosetteSep networks of unwanted blood cells were first formed using a cocktail of tetrameric complexes of antibodies against several blood cell markers (CD2, CD16, CD19, CD36, CD38, CD45, CD66b and glycophorin A). After rosette formation, blood samples were layered on a density gradient medium and centrifuged to separate the sample in different phases: plasma, cells of interest, and rosettes of unwanted blood cells. The cells of interest were enriched and collected on the upper layer of the density gradient medium for analysis; unwanted cells were discarded. Then, CTCs were detected using the DEPArray after labeling the enriched cells with a cocktail of antibodies against markers selected by the characterization of MCC cell lines: EpCAM, CK20, Pan CK, NSE, and CD45 (exclusion marker specific of leukocytes).

The DEPArray is a single-cell sorting technology system that uses cartridges with microfluidic chambers and dielectrophoresis cages to capture cells by charge affinity. According to the manufacturer’s instructions, cells were loaded in the micro-chamber of the cartridge that is then scanned automatically using up to six fluorescent channels. In this study, FITC was used to detect epithelial markers (EpCAM, CK20 and Pan CK), APC for NSE, and PE for CD45. Cells were then selected according to their phenotype and staining pattern as either single cells or as small pools of cells with the same phenotype. With this high-tech system, rare CTCs can be used for further experiments, such as MCPyV DNA detection in single CTCs (this study).

### Spiking experiments: dilution of cancer cell lines in blood from healthy donors

These experiments allowed mimicking blood samples of patients with MCC by spiking known and controlled numbers of MCC cells in blood samples of healthy donors obtained at the Établissement Français du Sang. Specifically, 100, 50, and 25 cells (MCCL-11 or MKL-1 cells) were added to 7.5 mL (CellSearch system) and 10 mL (RosetteSep-DEPArray workflow) of blood to evaluate the recovery rate and detection threshold of the two methods. MCCL-11 and MCCL-9 were suspended by pipetting, MKL-1 were dissociated using the Accumax solution (eBIOscience, ref. 00–4666–56). The real number of tumor cells added to each blood sample was confirmed by counting an aliquot of diluted cells under a microscope, and the recovery rate was corrected if required. The evaluation of the enrichment yield was determined by addition of calcein, a cell-permeant dye that labels viable cells and that allowed following the tumor cell number through all successive steps of the RosetteSep-DEPArray workflow.

### DNA preparation from single/pools of CTCs and from tumor biopsies

The genome of single CTCs sorted with the DEPArray was amplified with the Ampli1 WGA kit (MENARINI SILICON BIOSYSTEMS) following the manufacturer’s instructions. After genome amplification, the Ampli1 WGA Quality Control kit was used, as recommended by the supplier, to determine the DNA quality before further analyses.

MCC tissue biopsies of eight patients positive for CTCs were collected from different pathology laboratories (University Medical Centers of Montpellier and Nîmes; Hospitals of Alès, Avignon, Béziers and Perpignan; Inopath LaboSud, Inovie Group). Genomic DNA was extracted from these formalin-fixed paraffin-embedded (FFPE) samples with the QIAamp DNA FFPE Tissue Kit (QIAGEN) following the manufacturer’s instructions, and was stored at −20 °C until use.

### Single CTC sequencing

The *Ampli*1 LowPass Kit for Illumina platforms (MENARINI SILICON BIOSYSTEMS) was used for preparing low-pass whole genome sequencing libraries from five single CTC cells. Libraries concentrations were then normalized and sequenced with the MiSeq System (150 SR run mode). The obtained FASTQ files were aligned to the hg19 human reference sequence using Burrows-Wheeler Aligner, version 0.7.12 (BWA)^42^. Copy-number aberrations were identified using the Control-FREEC^43^ software (version 11.0). Ploidy level was automatically estimated by the MENARINI SILICON BIOSYSTEMS pipeline for each library, based on best fitting of the copy number profiles of the samples to the underlying copy number levels.

### MCPyV DNA detection

MCPyV DNA was detected by a real-time PCR assay using primers that target the VP2/3 region [MCPyV-2.0–4367 F (5′-GGCAGCATCCCGGCTTA-3′) and MCPyV-2.0–4399 R (5′-CCAAAAAGAAAAGCATCATCCA-3′)], and the dual-labeled probe MCPyV-2.0–4371-Prb (5′-FAM-ATACATTGCCTTTTGGGTGTTTT-BHQ1-3′), as already described by Bialasiewicz *et al*.^44^.

A qPCR targeting the C-term part of the large T antigen of MCPyV was investigated using [MCV_TAg_C_fw1 (5′-AGATGGTGCTGTAGCTG-3′) and MCV_TAg_C_bw1 (5′-AGGGAATCTCTTAGATTTGCC-3′) and the dual-labeled probe MCV_TAg_C_p1 (5′-6-FAM-TTTCCTCCTTGTATTGTTACTGCTAATGATTATTT-BBQ-3′)], as described by Schmitt *et al*.^45^

Investigation of MCPyV detection in FFPE DNA was done on normalized concentration at 40 ng/uL. For amplified DNA from single cells, the amount was not known, thus 5 uL of WGA product was used for the MCPyV detection. Positive samples were samples where the MCPyV signal was observed before the 40^th^ cycle of the qPCR.

### Blood collection from healthy donors and MCC patients

After signature of the informed consent by all participants in this study, peripheral blood samples were collected. All the experiments were performed in accordance with the relevant guidelines and regulations and were approved by the Bioethical Committee of the University Medical Center of Montpellier ‘IRB’ (No 2018_IRB_MTP_12-07). Blood samples from 19 patients with MCC followed at the Department of Dermatology (University Medical Center of Montpellier) between October 2017 and January 2019 were collected in Cell-Free DNA BCT tubes (STRECK) and CellSave tubes (MENARINI SILICON BIOSYSTEMS), then processed within 24 hours after blood collection using the RosetteSep-DEPArray workflow and the CellSearch system. In total, 28 blood samples were analyzed. Blood samples from three healthy donors have been provided by the Établissement Français du Sang.

### Statistical analysis

The correlation between clinico-pathological characteristics and CTC detection was analyzed using the Fisher’s exact test. Results were considered significant when *p* ≤ 0.05. Age, sex, American Joint Committee on Cancer (AJCC) tumor stage, absence/presence of distant metastases (M_0_/M_1_), number of organs with metastases and cancer antecedents were compared in patients with CTC detected by combining both complementary technologies and by using each method separately.

## Supplementary information

Supplementary Information.
