# Supplementary material for: Circulating Tumor Cell Detection and Polyomavirus Status in Merkel Cell Carcinoma
Source: Sci Rep. 2020 Jan 31;10:1612. doi: 10.1038/s41598-020-58572-9 (PMC6994658; doi:10.1038/s41598-020-58572-9)
Supplement: Supplementary file 1 — Supplementary Information. [file 41598_2020_58572_MOESM1_ESM.docx]

**Circulating Tumor Cell Detection and Polyomavirus Status in Merkel Cell Carcinoma**

Magali Boyer^1^, Laure Cayrefourcq^1^, Françoise Garima^1^, Vincent Foulongne^2^, Olivier Dereure^3^, Catherine Alix-Panabières^1┼^

^1^Laboratory of Rare Human Circulating Cells, University Medical Centre of Montpellier, 34093 Montpellier, France

^2^Pathogenesis and Control of Chronic Infections, University of Montpellier, INSERM, EFS, University Medical Centre, 34090 Montpellier, France

^3^Department of Dermatology, University Medical Centre of Montpellier and INSERM 1058 Pathogenesis and Control of Chronic Infections University of Montpellier, 34090 Montpellier, France

^┼^Corresponding author: Catherine Alix-Panabières, University Medical Centre of Montpellier, Laboratory of Rare Circulating Human Cells (LCCRH), IURC, 641, Avenue du Doyen Gaston Giraud, 34093 Montpellier Cedex 5, France. *Tel:* +33-4-1175-99-31; *Fax:* +33-4-1175-99-33; Email: [c-panabieres@chu-montpellier.fr](mailto:c-panabieres@chu-montpellier.fr).

**Supplementary Information**


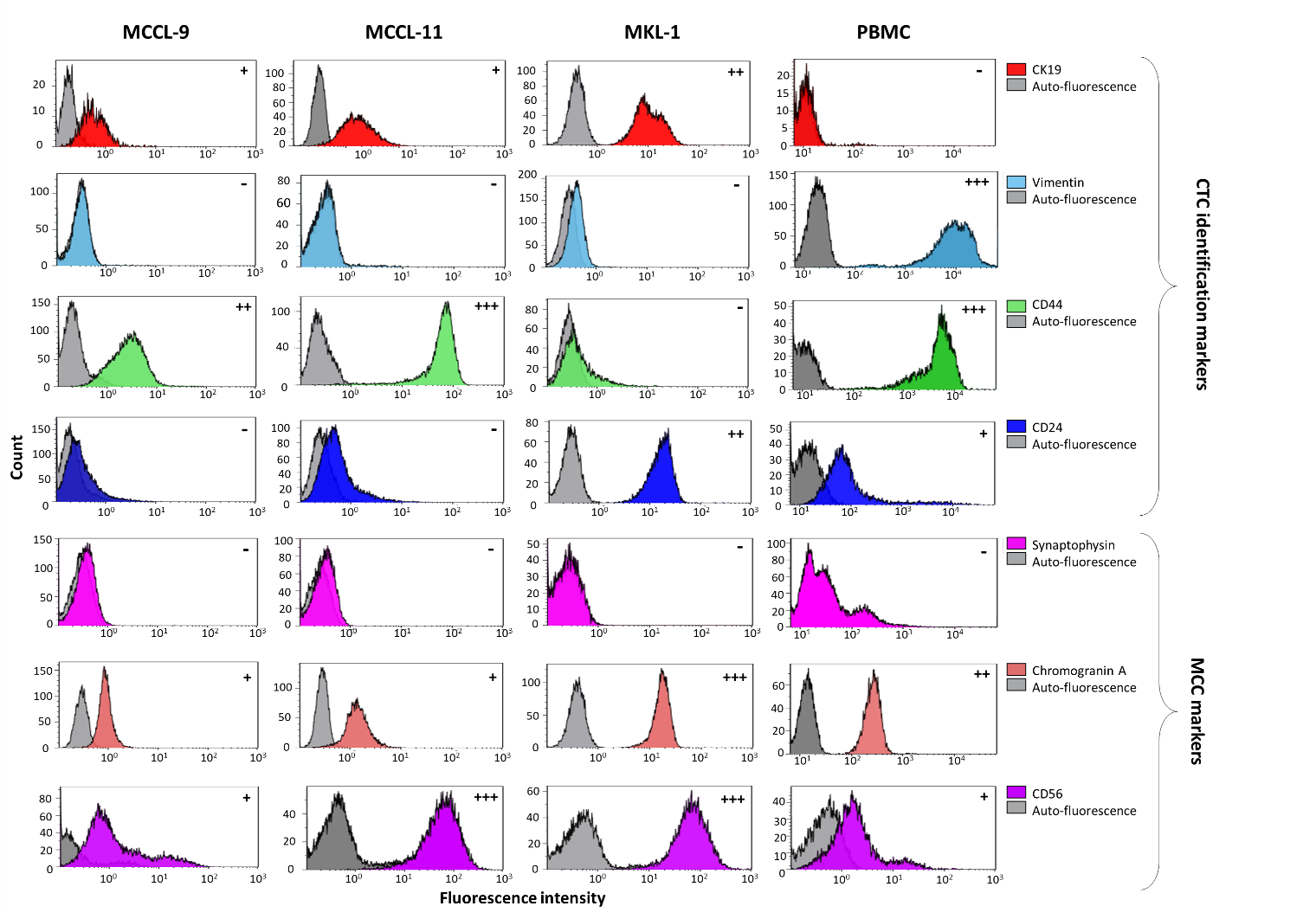


**Supplementary Fig. 1. Phenotypic characterization of MCC cell lines.** Specific CTC, leukocyte and MCC markers were used to characterize the MCCL-9, MCCL-11 and MKL-1 Merkel cancer cell lines and PBMCs as controls. The markers presented in this Fig. are those that have not been selected to detect CTCs in MCC. (+) represents a positive marker, and the number of (+) indicates the signal intensity. (–) represents a negative marker. Grey represent the auto-fluorescence of the MCC cell lines (negative control). All these markers were also tested on PBMCs to determine which markers can be used to discriminate CTCs from normal blood cells. *Abbreviations:* CK: Cytokeratin., CD: Cluster Differentiation.


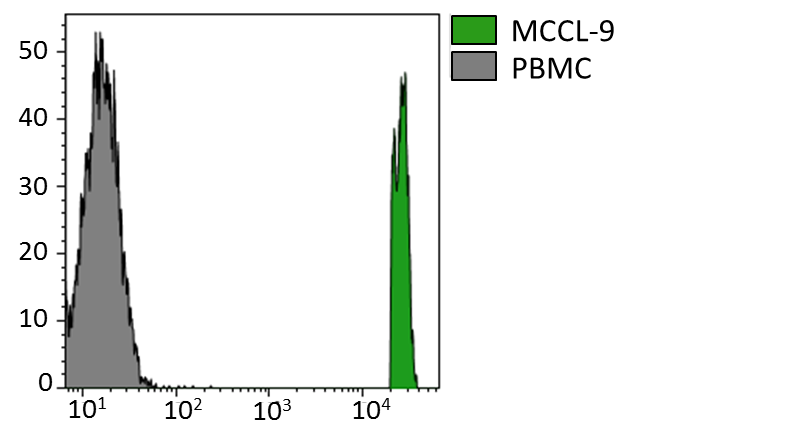


**Supplementary Fig.** **2. EpCAM expression by the MCCL-9 cell line and PBMCs.**

*Abbreviations:* EpCAM: Epithelial Cell Adhesion Molecule.


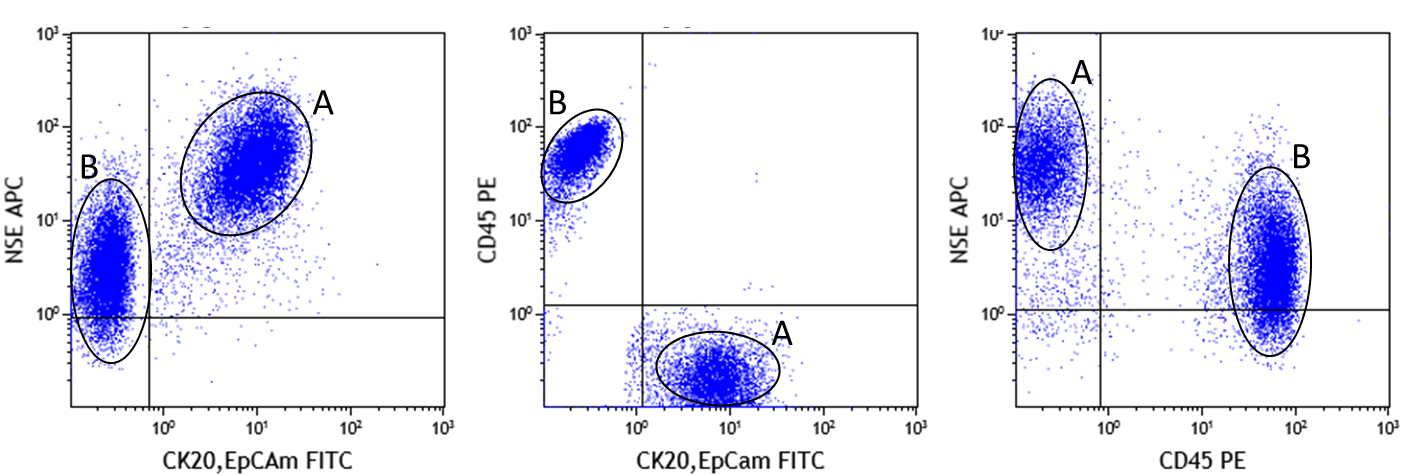


**Supplementary Fig. 3. Discrimination of tumor cells and PBMCs using flow cytometry.** A mix of MCCL-11 and PBMCs was labelled with the cocktail of antibodies described in Fig. 1. MCCL-11 population is designated by “A” and PBMCs population by “B” in each graph.

| **Antibodies (Abs)** | **Conjugated** | **References** | **Suppliers** | **Dilutions** |
| --- | --- | --- | --- | --- |
| Chromogranin A | FITC | FCMAB329F | Millipore | 1/20 |
| CD24 | PE | 130-095-953 | Miltenyi | 1/50 |
| CD44 | FITC | J.173 | Bekman Coulter | 1/20 |
| CD45 | PE | 130-113-118 | Miltenyi | 1/14 |
| CD56 | APC | 130-113-867 | Miltenyi | 1/20 |
| CK19 | FITC | 61029 | Progen | 1/50 |
| CK20 | FITC | ab189087 | Abcam | 1/14 |
| PanCK (8,18,19) | FITC | 130-080-101 | Miltenyi | 1/20 |
| EpCAM | FITC | 130-080-301 | Miltenyi | 1/33 |
| Neuro-Specific Enolase (NSE) | Alexa 647 | ab199829 | Abcam | 1/14 |
| Synaptophysin | Alexa 647 | bs-0121R-AF647 | Bioss | 1/100 |
| Vimentin | PE | 562337 | BD Bioscience | 1/50 |

**Supplementary Table 1: Listing of the antibodies used for MCC cell line characterization**
